# Micro-CT Study of Mongolian Gerbil Humeral Bone After Prolonged Spaceflight Based on a New Algorithm for Delimitation of Long-Bone Regions

**DOI:** 10.3389/fphys.2021.752893

**Published:** 2021-12-07

**Authors:** Yuri S. Krivonosov, Victoria I. Gulimova, Alexey V. Buzmakov, Denis A. Zolotov, Alessia Cedola, Inna Bukreeva, Victor E. Asadchikov, Sergey V. Saveliev

**Affiliations:** ^1^Laboratory of X-ray Reflectometry and SAXS, Federal Scientific Research Centre “Crystallography and Photonics” of Russian Academy of Sciences, Moscow, Russia; ^2^Laboratory of Nervous System Development, Federal State Budgetary Institution “A. P. Avtsyn Research Institute of Human Morphology”, Moscow, Russia; ^3^Institute of Nanotechnology, CNR, Rome Unit, Rome, Italy; ^4^X-ray Optics Laboratory, P. N. Lebedev Physical Institute of the Russian Academy of Sciences, Moscow, Russia

**Keywords:** Mongolian gerbil, micro-computed tomography (μCT), micro-CT, humerus, weightlessness, microgravity, Foton-M3

## Abstract

The Mongolian gerbil displays unique physiological and anatomical features that make this species an attractive object for biological experiments in space. However, until recently, the Mongolian gerbil has remained a novel, mostly unstudied animal model in investigating bone loss in weightlessness (G_0_). After 12 days of orbital Foton-M3 mission, the humerus of Mongolian gerbils has been studied here *via* micro-computed tomography (micro-CT) to quantify bone morphometric parameters. The samples from the flight group, delayed synchronous ground-control group, and basal control group were investigated, and main morphometric parameters were reported in the article. The accurate selection of a region of interest is an essential step for a correct assessment of bone parameters. We proposed a new, easy and efficient method for delimiting the bone’s basic regions in the humerus. It is based on quantitative estimation of X-ray attenuation in the cortical bone as a function of humerus bone length. The micro-CT analysis of the basic bone regions revealed a difference in bone morphometric parameters between the flight and control gerbils. The most significant bone loss was observed in the cortical part of the proximal humeral zone in the flight group. No statistically significant changes of volume fraction in the cancellous tissue of proximal and distal epiphyses and metaphyses were observed. A statistically significant increase in both cancellous bone volume and bone X-ray attenuation in the flight group was detected in the proximal part of the diaphyses. We assume that enhanced calcium deposition in the diaphyseal cancellous tissue occurred due to a bone response to G_0_ conditions.

## Introduction

After 12 days of orbital Foton-M3 mission, the humerus of the Mongolian gerbil has been studied by us *via* X-ray micro-computed tomography (micro-CT) to identify bone morphometric parameters. The negative impact of spaceflight on the human and animal skeleton is well known and remains a critical issue until nowadays (Clément, 2011; Nagaraja and Risin, 2013; Berg-Johansen et al., 2016). Studies of a human bone system in G_0_ detected an average bone loss of 1–2% per month, during the flight at the Russian “Mir” spacecraft and International Space Station (ISS) (Lang et al., 2004; Clément, 2011; Sibonga et al., 2015). Most of the current publications reported cancellous tissue loss in the weight-bearing bones (Lang et al., 2004, 2017; Bloomfield et al., 2011; Nagaraja and Risin, 2013; Gerbaix et al., 2017; Maupin et al., 2019; Tominari et al., 2019). Until now, in human research programs, the knowledge about the G_0_ impact on bone is significantly limited by the lack of diversity in race, gender, and age. Moreover, the study is restricted by human examination without doing a biopsy. Therefore, the appropriate animal model has become increasingly important for space-based biomedical research.

Mongolian gerbil (*Meriones unguiculatus*) is a member of the rodent family. Rodents are the animal models most commonly used in G_0_ research. However, until recently, only mice and rats have long been used to study principle biological processes in space (Cancedda et al., 2012; Santucci et al., 2012; Andreev-Andrievskiy et al., 2014; Ronca et al., 2019), including G_0_-associated bone loss (Tavella et al., 2012; Blaber et al., 2013; Lloyd et al., 2015; Berg-Johansen et al., 2016; Gerbaix et al., 2017). In particular, experiments with Wistar rats (*Rattus norvegicus*) on biosatellites of the Bion series No. 1-6 (1973–1986) provided a large amount of data on morphological, histochemical, and biochemical changes induced by weightlessness, artificial gravity, and the combined effects of weightlessness and ionizing radiation (Il’in et al., 1989). In recent researches, mice increasingly have been used for the analysis of structural and molecular genetic changes that occur in the bones of the skeleton under G_0_ (Giuliani et al., 2018; Endicott and Fitzgerald, 2019; Tominari et al., 2019).

Although the rodents are the most used species in G_0_ research, the Mongolian gerbil is a relatively new and insufficiently studied object in space-related investigations. Only recently, *de novo* sequencing and initial annotation of their genome have been reported (Zorio et al., 2019). The 30-day orbital experiment with gerbils at the Bion-M1 unmanned spacecraft in 2013 resulted in the death of animals for technical reasons (Sychev et al., 2014), other studies of these animals in G_0_ have not been carried out, and it is unlikely that such experiments will be possible soon.

The gerbils are representative of the fauna of desert rodents. Among them, there are species with daytime and nighttime activity, and territorial (i.e., protecting their habitats) and non-territorial behavior (Gromov, 2000). Since gerbils are usually more active than rats, they are likely more sensitive to the lack of normal dynamic and static loading in weightlessness (Durnova et al., 2008). Gerbils secrete only a small amount of concentrated urine since, due to the special structure of the kidneys, most of the water is reabsorbed in the ascending sections of Henle’s nephron loops (Kaplanskiǐ et al., 2008). Therefore, the urinary calcium excretion in G_0_ can be reduced. On the other hand, a potential increase in fecal calcium excretion should be taken into consideration (Durnova et al., 2008).

Gerbils, due to their unique features, have proved to be an appropriate animal model for studying many human disturbances and diseases. Generally, gerbils have been used in research involving cerebral ischemia, parasitology, infectious diseases, epilepsy, brain development, behavior, and hearing (Batchelder et al., 2012). Besides, the Mongolian gerbils display several features that make them an attractive object for experiments in space. The bodyweight of the adult gerbil (40–50 g) is about twice that of a mouse but almost ten times less than that of a rat. Moreover, Mongolian gerbils are low-maintenance diurnal animals due to adaptation to arid conditions, which allows using an easier and compact habitat unit and life support system during the spacecraft mission (Il’in et al., 2009). A gerbil’s life span in a vivarium is about four years. They reproduce well in captivity. The gerbils are easy to handle and usually calm during manipulations. They can withstand the temperature extremes and longtime water deprivation in the presence of succulent feed (Batchelder et al., 2012). The macro and microscopic structures of internal organs in gerbils, rats, and mice are similar (Kaplanskiǐ et al., 2008). At the same time, the gerbil retinal structure (Govardovskii et al., 1992) and sensitivity to the sounds of low frequency (Ryan, 1976) are human-like. As a model translational to humans, recently, gerbils have proved highly suitable to study spaceflight-related adaptation effects on calcium metabolism. In particular, it was reported the results of the investigation on the proximal epiphyses of the tibia after the 12-day flight experiment at the Foton-M3 spacecraft (Durnova et al., 2008). The humerus of Mongolian gerbils is a novel, mostly unstudied model in space experiments. To our best knowledge, no research has been reported on the cortical or cancellous bone in the proximal epiphysis, diaphysis, and distal humeral epiphysis of gerbils after a long-term space flight experiment.

We study here the humerus of gerbils *via* micro-CT. The three-dimensional (3D) reconstructed image has been used for the delimitation of the basic regions in the bone and volumetric assessment of the bone morphometric parameters. Accurate selection of a region of interest (ROI) is an essential step for the correct bone morphometry. Despite numerous studies and a large number of segmentation algorithms, until recently, no easy and efficient numerical method allowing the accurate definition of the boundaries between the main regions of the long bone has been proposed. In particular, it concerns the boundaries delimitating the diaphysis and metaphyses.

A lack of clear criteria for ROI selection considering the general gross anatomy of the bone makes it difficult to compare and reproduce the obtained results. Most publications on bone researches have been dedicated to the analysis of local ROI in different bone regions (Canciani et al., 2015; Keune et al., 2015; Berg-Johansen et al., 2016) while the general information about the gross anatomy of the bone was usually neglected (Yamashita et al., 2000; Almeida et al., 2006).

Authors of a recent study (Williams et al., 2020) used a micro-CT-based 2D morphometric analysis of long bone to investigate trabecular and cortical bone characteristics as a function of bone length. This research has provided a more systematic approach to ROI selection than the methods based on anatomical landmarks. Nevertheless, it does not allow an accurate delineation of the basic regions in the bone.

We aimed in our research to develop a new advanced numerical algorithm for a micro-CT based study of the long bone, which gives key criteria to delimitate the basic regions in the bone, such as proximal/superior epiphysis & metaphyses (proximal EM-zone), diaphysis, and distal/lower epiphysis & metaphysis (distal EM-zone). The proposed method provides a basis for accurate morphometric analysis of both cortical and cancellous tissues in each bone region. The algorithm is based on the analysis of X-ray attenuation in the cortical tissue within the adjusted cross-sections of the bone. We have found a significant variation of the attenuation coefficient in the proximal and distal EM-zones, while the attenuation varied slightly within the diaphysis. Quantitative estimation of the cortical bone attenuation allowed us to delimitate the principal regions of the humerus.

We have developed our method as a useful scientific tool for bone research that could overcome the issues with a standard definition of boundaries between the main regions of the long bone in different samples. The algorithm would provide a basis for subsequent development of generic semiautomatic or automatic tools for the bone regions standard segmentation in both humans’ and animals’ long bones. The generic method would allow different authors to present their results more comparably way, facilitate understanding between the scientific groups and increase research output in this area (Giuliani et al., 2018). We note that the obtained results would be useful not only in spaceflight-related science but also in conventional biological and medical research.

## Materials and Methods

### Experimental Conditions

The difference in morphometry of the humerus bones between the space and control groups of Mongolian gerbils (*Meriones unguiculatus*; Milne-Edwards, 1867) after a 12-day orbital flight (Foton-M3 satellite mission, September 14–26, 2007) has been investigated *via* micro-CT. The experimental groups consisted of sexually mature male gerbils, 4–4.5 months of age [the age of sexual maturity is 9–12 weeks for both sexes (Batchelder et al., 2012)], with the average weight of all the animals in the groups of 56.2 g. Twenty-seven gerbils humerus bones of three groups of gerbils have been studied. Specifically, 12 samples of the flight group, 11 samples of the delayed synchronous ground-control experiment, and 4 samples of a basal control group were investigated.

The unit of selection of each group was the whole family (not individual animals, as in the case of mice and rats) because gerbils are social animals. Each group of the experiment was formed of pups from the same parents (Il’in et al., 2009). All families were selected from the same subpopulation of the State Scientific Center of the Russian Federation Institute of Biomedical Problems of the Russian Academy of Sciences (SSC RF–IBMP RAS) vivarium gerbils with genetic similarity. No statistically significant differences in bone volume, bone volume fraction, and bone X-ray attenuation between the basal and delayed synchronous control groups were found. The animals of these two groups were united into one common control group. In addition, since the parameters of sample No.3 from the basal control group significantly differed from the parameters of all groups, this sample was excluded from the analysis.

Onboard the spacecraft, gerbils were housed in a space animal enclosure module “CONTUR-L,” which was developed to provide an autonomous system of life support in the “Foton-M3” mission (Ilyin et al., 2008; Soldatov et al., 2009). The satellite environmental control systems of the “Foton-M” satellite series provided only centralized thermal control.

During the flight, the animals were maintained in a cage with dimension 12.5 cm^3^ × 25.5 cm^3^ × 37.0 cm^3^, made of steel wire mesh with 8 × 8 mm^2^ cells. A constant laminar flow of a gaseous medium with a linear velocity of 0.3 m/s passed through the cage in the up-down direction. Respiratory gas impurities and carbon dioxide was continuously removed by an absorption cartridge with lithium hydroxide, activated carbon, and catalysts. Waste removal was carried out once a day using a tape mechanism. The cage was illuminated by a panel of yellow LEDs in the day and night mode as 12:12 h. The module was equipped with a digital video recorder that provided continuous video recording during daylight hours (Il’in et al., 2009).

The gerbils of the spaceflight group were housed in the “CONTUR-L” module two days before the flight. The briquette food consisted of natural ingredients (cereals, dried fruits, feed additives, and binders with a moisture content of 20%) (Soldatov et al., 2008). The animals were automatically supplied two feed briquettes with a weight of 55 g once daily by food dispensers. On September 23, 2009, there was a failure of one feeding dispenser, and the gerbils of the flight group were on a half food diet until September 25, 2009. The median flight temperature was +27.4°C. Animals of the three groups were euthanized one day after the end of the appropriate experiment. The average weight of animals was about 51.6 g before the flight and about 37.1 g after the flight.

The delayed synchronous control experiment was carried out in the State Scientific Center of the Russian Federation Institute of Biomedical Problems of the Russian Academy of Sciences (SSC RF–IBMP RAS) with a delay of 2 days concerning the flight schedule. According to the results of telemetry, the experiment reproduced all main spaceflight conditions, except weightlessness. Due to a failure in the supply of telemetry system information, animals from the synchronous group were fed half of the daily food with a delay of 4 days compared to the flight group (from the 9th day instead of the 5th day). The median temperature in the delayed synchronous control experiment was +26.9°C. The average bodyweight of the animals was about 63.4 g before the experiment and about 54.2 g after the experiment.

An experiment with a basal control group was carried out in the vivarium of the SSC RF–IBMP RAS. The animals during 14 days were housed in standard vivarium cages comparable in size to the in-flight cage, then they were euthanized on the day of launch. The basal group was fed the same amount and quality of food as it was planned for the flight group (the failure of the feeding dispenser was not imitated in this group). The animals were deprived of water for 14 days under flight conditions. The median temperature in the basal control experiment was +23.0°C. The average bodyweight of the animals before the experiment was about 53.7 g after the experiment was about 50.5 g.

#### Ethics Statement

The animal study was reviewed and approved by the Biomedicine Ethics Committee of the Russian Federation State Research Center—Institute of Biomedical Problems, Russian Academy of Sciences/Physiology Section of the Russian Bioethics Committee of Russian Federation National Commission for UNESCO/, minute No. 206 from September 7, 2007.

### Samples Preparation

The forelimbs of clawed (Mongolian) gerbils were fixed in 4% neutral buffered formalin (pH 7.2–7.4). Following water rinsing after formalin fixation, humerus bones were separated from the soft tissues, and dried and stored at room temperature until they were scanned by laboratory X-ray micro-CT. We studied a total of 27 samples. Namely, 12 flight samples, 4 samples from the basal control, and 11 samples from the delayed synchronous control were analyzed.

### Micro-CT Setup

Micro-CT setup has been developed at the Institute of Crystallography of Federal Scientific Research Centre “Crystallography and Photonics” of the Russian Academy of Sciences (Buzmakov et al., 2018). An X-ray tube with a molybdenum anode was used as an X-ray source. A pyrolytic graphite crystal monochromator was tuned to a photon energy of 17.5 keV. CCD detector (XIMEA xiRay11) used in the experiment had a pixel size of 9 × 9 micron^2^. Tomographic images were reconstructed from 400 projections acquired in parallel scanning geometry over an angular range of 200 degrees with increments of 0.5 degrees.

### Tomographic Reconstruction and Image Processing

**FIGURE 1:**
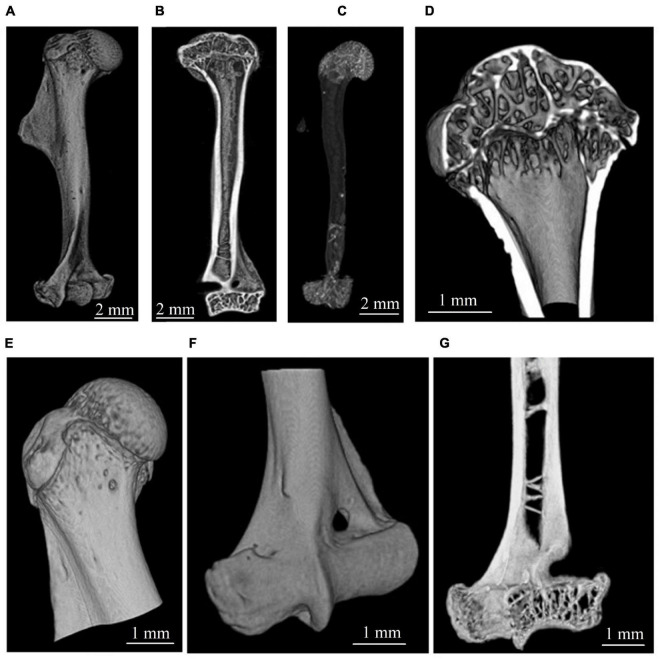
Micro-CT visualization of gerbil’s humerus. **(A,B)** Representative image of the whole humerus. **(C)** Segmented total cancellous compartment of humerus. **(D,E)** Bone structure in the proximal zone. **(F,G)** Bone structure in the distal zone.

The tomographic reconstruction was performed with the CGLS algebraic method (Van Aarle et al., 2016; Buzmakov et al., 2019). Micro-CT reconstructed images of the whole humerus and its compartments are shown in Figure 1. Three-dimensional visualization of image stacks was performed with the open-source image processing software ImageJ/Fiji (Schindelin et al., 2012).

**FIGURE 2:**
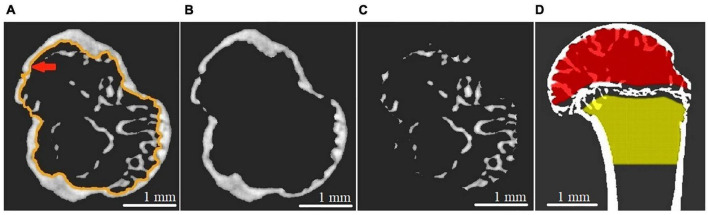
Transverse CT section of the proximal epiphysis of gerbil’s humerus scanned with micro-CT setup at 17.5 keV energy and 9 × 9 micron^2^ pixel size. **(A)** The image after the binarization procedure. The red arrow indicates the endocortical surface line. **(B)** Segmented cortical bone. **(C)** Segmented cancellous bone. **(D)** Representative image of segmented epiphysial (red) and metaphyseal (yellow) subcortical volume, taking the epiphyseal plate as an anatomical reference.

The reconstructed micro-CT slices were used for a virtual segmentation of humerus bone tissue (Asadchikov et al., 2012). The segmentation was performed with the thresholding binarization method. A global threshold in all tomographic images was found using Otsu’s method (Otsu, 1979) to exclude the soft tissue from the analysis. The segmentation of cortical and cancellous bone was performed in semiautomatic mode using morphological operations (dilation, erosion, closing, etc.) described in Gonzalez et al. (2004). We relied on the expert-biologists valuation to check the quality of the segmentation. The representative images of cortical and cancellous bone tissue in the proximal epiphysis of the humerus bone are shown in Figures 2A–C.

**FIGURE 3:**
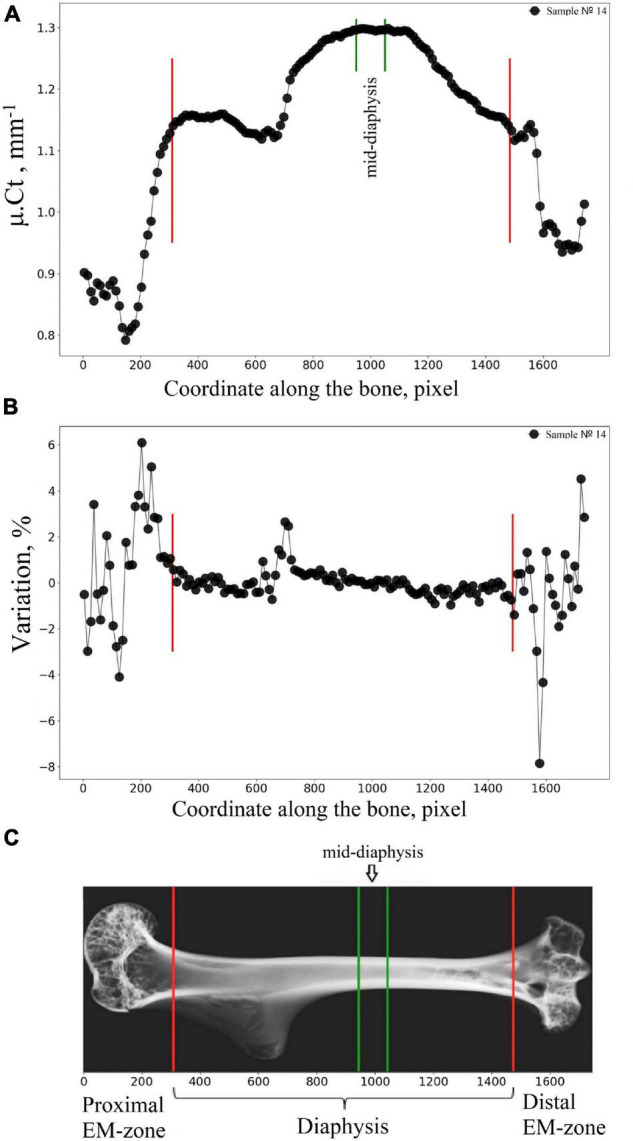
Segmentation of the humerus into the diaphysis, proximal EM-zone, and distal EM-zone with the developed algorithm. **(A)** Linear attenuation coefficient of the cortical bone (average over 100 microns thick transverse section of the humerus) vs. the coordinate of the section along the humerus (bone longitudinal coordinate). **(B)** The “Variation” of the attenuation coefficient with respect to the bone longitudinal coordinate. **(C)** Representative micro-CT image of the segmented zones in gerbil’s humerus. Red lines indicate the boundaries between the proximal EM-zones and the diaphysis and between the distal EM-zones and the diaphysis. Green lines outline the mid-diaphysis region.

We developed and applied here the advanced algorithm dedicated to a semiautomatic segmentation of the humerus into the diaphysis, proximal EM-zone, and distal EM-zone. Micro-CT image of cortical bone was virtually transected into 100 μm thick sections, and the mean attenuation value μ.*Ct*_*i*_ (*i* = *1, 2*,…*N*) of cortical bone in each section was calculated (Figure 3A). Subsequently, the *variation* parameter (see Figure 3B) was found as a difference of the mean attenuation values μ.*Ct*_*i*_ between the adjacent transverse sections of the bone expressed as a percentage (*Variation*_*i*_ = 100*(μ.*Ct*_*i*_−μ.*Ct*_*i*−1_)/μ.*Ct*_*i*−1_,[%]). A border between two neighbor transverse sections with a *variation* of more than 1% was considered as a borderline (red lines in Figure 3) between the regions of the bone. The boundaries location between the main regions of the humerus bone found with our algorithm was within the following limits. In the proximal part of the humerus, the offset (the distance along the bone’s long axis from the edge of the sample to the boundary between the metaphysis and the diaphysis), expressed as a percentage of sample length, varied within 16.5–21.5% with an average value of 18.5%. In the distal part, the offset varied within 11.6–17.9% with an average value of 15.6%.

### List of Humerus Bone Morphometric Parameters

The morphometric parameters: sample length (Sa.Le), total volume (TV: the volume of the whole examined sample), bone volume (BV), normalized volume index (BV/TV), and linear attenuation coefficient of bone (μ.B) were calculated for each sample of the humerus. The linear attenuation coefficient of cortical bone (μ.*Ct*) and cancellous bone (μ.*Cn*), cortical bone volume normalized by total volume (Ct.BV/TV: cortical bone volume fraction), cancellous bone volume normalized by subcortical volume (Cn.BV/Sc.V: cancellous bone volume fraction) were calculated for regions of interest (proximal EM-zone, distal EM-zone, and diaphysis). It should be noted that by the subcortical volume (Sc.V), we mean the volume limited by the endocortical surface (see Figure 2A). To calculate the morphometric parameters mentioned above, the entire cortical and cancellous bone compartments were selected in each ROI. The values of linear attenuation coefficients were calculated as median values of bone attenuation.

Model-independent algorithm (Hildebrand and Ruegsegger, 1997; Hildebrand et al., 1999) was used to calculate the bone morphometric parameters such as mean trabecular thickness (Tb.Th), mean trabecular separation (Tb.Sp), mean trabecular number (Tb.N) in the distal epiphysis. The entire cancellous compartment of the distal epiphysis (110 slices, 990 microns) was considered. In the calculation of the trabecular bone parameters (Tb.Th, Tb.Sp, Tb.N) the metaphyseal part of the bone was neglected because the volume fraction of trabecular bone in this part was relatively small for an accurate estimation of average trabecular separation (Tb.Sp) and average trabecular number (Tb.N). The results of calculations of the trabecular bone morphometric parameters in accordance with the segmentation of the proximal epiphysis and metaphysis, taking the epiphyseal plate (Figure 2D) as an anatomical reference are presented in Supplementary Section 1.

One hundred slices (900 microns) of the cortical bone were selected in the humerus mid-diaphysis to calculate the next parameters: average total cross-sectional area (Tt.Ar), average cortical bone area (Ct.Ar), cortical area fraction (Ct.Ar/Tt.Ar) and average cortical thickness (Ct.Th). Average cortical thickness (Ct.Th) was calculated using distance-transform methods described in Hildebrand and Ruegsegger (1997). The abbreviations used in this work as well as the methodology of calculation correspond to Bouxsein et al. (2010) and Dempster et al. (2013). Morphometric parameters of the humerus were measured using Python. Parameters Tb.Th, Tb.Sp, Tb.N, Ct.Th were calculated with the Python Toolkit “Porespy” (Gostick et al., 2019).

### Statistical Analysis

The differences in the mean value of tested parameters of the humerus between the control and flight groups are presented as a percentage, while the value of the parameters in the control group was taken as 100%. The Shapiro–Wilk test was used to detection of normally distributed values. Comparisons between the control and flight groups were performed using Welch’s *t*-test for normally distributed values and Mann–Whitney *U*-test for non-normally distributed values. Spearman correlation test was used to investigate the correlation between the post-flight body weight of animals and the basic morphometric parameters of the humerus bone. Differences were considered statistically significant at *p* < 0.05 for all statistical tests. Statistical analysis was performed using Python “scipy.stats” package (Virtanen et al., 2020).

## Results

All gerbils survived the flight and landed in good health. Weight loss was observed in all experimental groups. An average decrease in body weight was 28.3, 12.9, and 5.9% in the flight group, delayed synchronous group, and the basal control group, respectively. No statistically significant correlation was found between the post-flight bodyweight of the animals and the morphometric parameters of the humerus bone (BV, BV/TV, μ.B) in all groups, excluding the volume of the whole examined sample (TV) in the control group (the Spearman’s correlation coefficients are presented in Supplementary Section 3).

**FIGURE 4:**
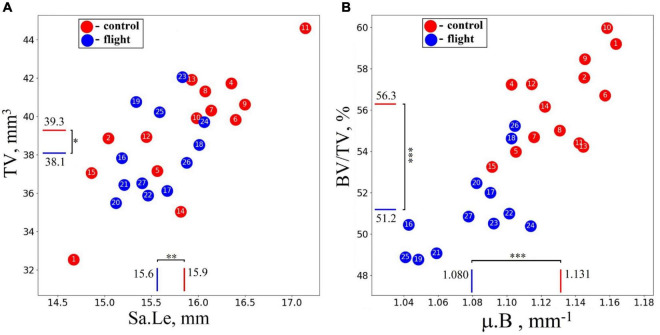
Morphometrical parameters of the humerus in the control group (red circles) and spaceflight groups (blue circles) were obtained with micro-CT imaging for the whole sample. **(A)** No statistically significant difference was detected in the total volume (TV) and sample length (Sa.Le) of the bone. **(B)** Bone volume fraction (BV/TV) was reduced by 9.1%, and the linear attenuation coefficient (μ.B) decreased by 4.6% in the humerus of the flight group compared with the control group. The red and blue lines indicate the mean values of the parameters in the control and flight groups, respectively (*p = 0.264, ^**^p = 0.178, ^***^p < 0.001).

A high-resolution micro-CT setup has been used for a quantitative assessment of the morphometric bone parameters in the humerus of the Mongolian gerbils. The terms “decrease” and “increase” throughout the text refer to the difference in morphometric parameters of the humerus between the groups. To evaluate the morphometry of the animal’s skeleton, the sample length (Sa.Le) and total volume (TV) of the humerus bone were estimated. No statistically significant difference in these parameters was detected between the flight and control groups (see Figure 4A). However, bone volume fraction (BV/TV) and linear attenuation coefficient (μ.B) were lower in the flight group than in the control group by 9.1 and 4.6%, respectively (Figure 4B).

**FIGURE 5:**
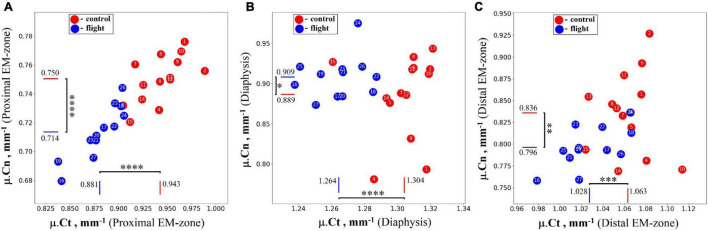
The linear attenuation coefficient of cancellous (μ.*Cn*) and cortical (μ.*Ct*) bone in the humeral proximal EM-zone, distal EM-zones, and diaphysis of control (red circles) and flight (blue circles) groups. **(A)** The proximal EM-zone—the attenuation coefficient in the cancellous bone decreased by 4.9%, and in the cortical bone decreased by 6.6% after the space flight. **(B)** The diaphysis—there was no statistically significant difference in the attenuation coefficient in the cancellous bone; the attenuation coefficient in the cortical bone decreased by 3.0% in the flight group. **(C)** The distal EM-zone—the attenuation in the cancellous bone decreased by 4.7%, in the cortical bone decreased by 3.4% in the flight group. The red and blue lines indicate the mean values of the parameters in the control and flight groups, respectively (*p = 0.738, ^**^p = 0.012, ^***^p = 0.002, ^*⁣*⁣**^p < 0.001).

Intergroup comparisons indicated the post-flight reduction in the mean value of the linear attenuation coefficient in cancellous and cortical parts of the proximal EM-zone by 4.9 and 6.6%, respectively (Figure 5A). A comparable but slightly smaller decrease was revealed in the distal EM-zone. The X-ray attenuation in the cancellous bone tissue decreased by 4.7% and in the cortical bone by 3.4% after the space flight (Figure 5C). In the diaphysis, no statistically significant differences in the trabecular bone X-ray attenuation were detected, but in the cortical part of the diaphysis, the attenuation decreased by 3.0% (Figure 5B).

**FIGURE 6:**
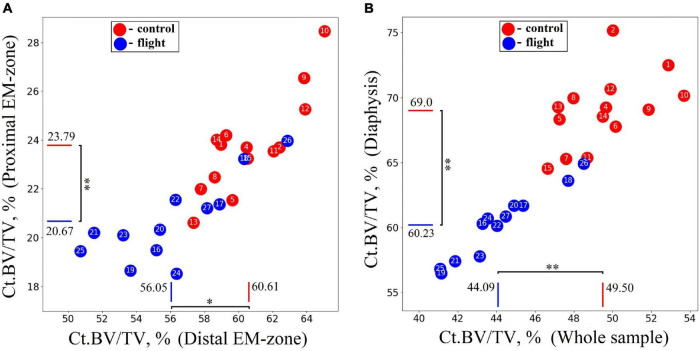
Comparative data analysis of the cortical bone volume fraction (Ct.BV/TV) in each humerus bone region in the control group (red circle) and flight group (blue circle). **(A)** Cortical bone volume fraction reduced by 13.1% in the proximal EM-zone; and by 7.5% in the distal EM-zone after the space flight. **(B)** Cortical bone volume fraction reduced by 12.7% in the diaphysis and by 10.9% in the whole humerus bone after the space flight. The red and blue lines indicate the mean values of the parameters in the control and flight groups, respectively (*p = 0.001, ^**^p < 0.001).

The research indicates a post-flight overall cortical bone loss in the humerus. Quantitative estimation of the cortical bone volume fraction Ct.BV/TV in each region of the bone shows an average decrease of 13.1% in the proximal EM-zone, 7.5% in the distal EM-zone (Figure 6A), and 12.7% in the diaphysis (Figure 6B). The average reduction of the cortical bone volume fraction within the whole humerus bone was 10.9% (Figure 6B).

**FIGURE 7:**
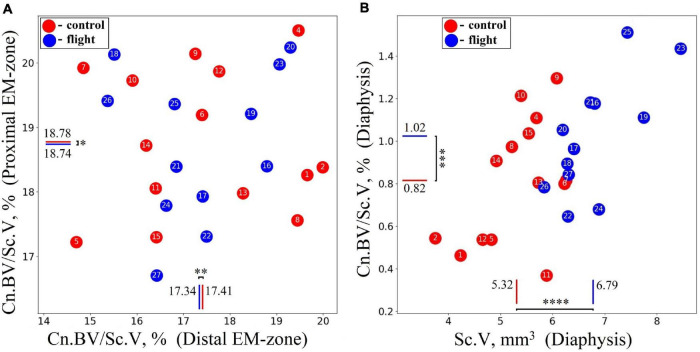
Comparative data analysis of the cancellous bone volume fraction (Cn.BV/Sc.V) of the humerus in the control group (red circle) and flight group (blue circle). **(A)** There was no statistically significant difference of Cn.BV/Sc.V in the proximal EM-zone and distal EM-zone. **(B)** In the diaphysis was observed a tendency (*p* = 0.073) to increase cancellous bone volume fraction (Cn.BV/Sc.V) by 25.6%; subcortical volume (Sc.V) increased by 27.7% in the diaphysis. The red and blue lines indicate the mean values of the parameters in the control and flight groups, respectively (*p = 0.937, ^**^p = 0.911, ^***^p = 0.073, ^*⁣*⁣**^p < 0.001).

The cancellous bone presented a different pattern. No statistically significant differences between the groups were found in the cancellous bone volume fraction (Cn.BV/Sc.V) within proximal and distal EM-zones (Figure 7A). On the other hand, a tendency (*p* = 0.073) to increase the value of Cn.BV/Sc.V by 25.6% was observed in the diaphysis of the flight group (Figure 7B) compared to the control.

Accordingly, the intergroup difference in bone parameters was found within all regions of the humerus. The maximum difference was observed in the cortical part of the bone. The tendency to increase the volume of the cancellous tissue during the flight was detected in the diaphysis. This may be explained both by bone minerals excretion from the humerus through the circulatory system and the redistribution of the minerals in the bone matrix.

**FIGURE 8:**
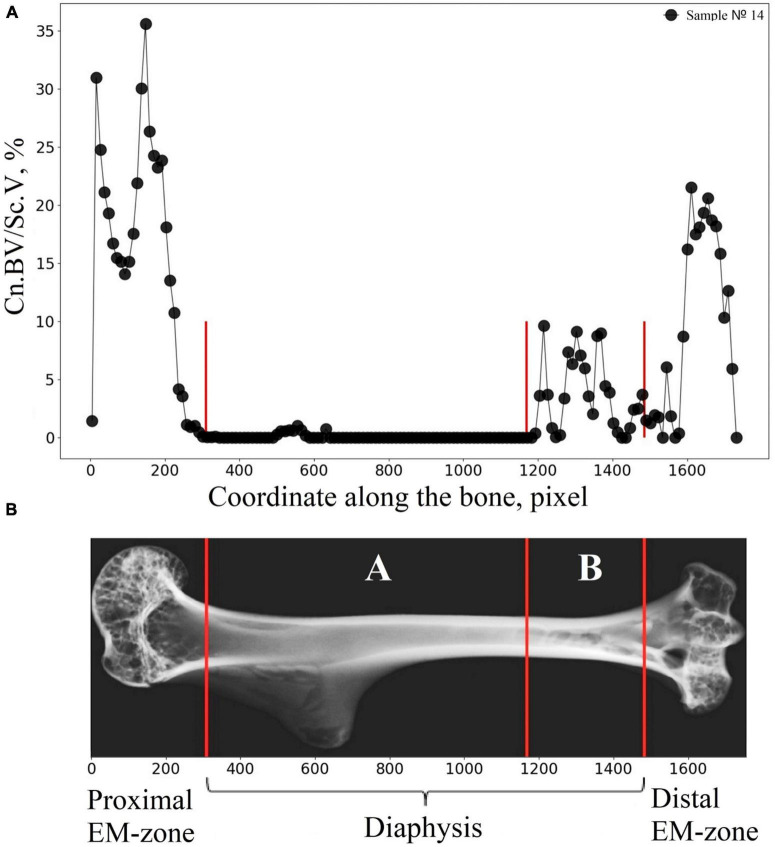
Illustration explaining the procedure of the bone segmentation with the developed algorithm. **(A)** The lengthwise change of the cancellous bone volume fraction (Cn.BV/Sc.V) along the humerus bone axis. **(B)** Representative micro-CT image of the segmented regions in gerbil’s humerus. Segmented regions in the diaphysis are indicated by white letters A and B. Red lines indicate the borderline of the segmented regions.

We noted that in the diaphysis, the cancellous bone volume fraction (Cn.BV/Sc.V) had a high standard deviation value for all studied groups. A large spread in values (Cn.BV/Sc.V) can be explained both by intraspecific individual variability in trabecular bone morphology and small volume of the cancellous bone tissue in the diaphysis (about 2% of the entire cancellous bone in the humerus). Therefore, the alterations in the diaphyseal cancellous bone required additional analysis. Accordingly, we investigated the lengthwise change of the cancellous bone volume fraction (Cn.BV/Sc.V) along the humerus. To achieve this, the cancellous bone was virtually segmented across the axis of the bone into 100 μm thick sections. The result presented in Figure 8A shows an extremely uneven distribution of the cancellous bone volume fraction in the diaphyseal subcortical region. A significant amount of the cancellous bone was observed in the site next to the distal EM-zone (Figure 8B), while a large subcortical area of the diaphysis next to the proximal EM-zones (Figure 8B) contained the cancellous bone only as an occasional inclusion.

**FIGURE 9:**
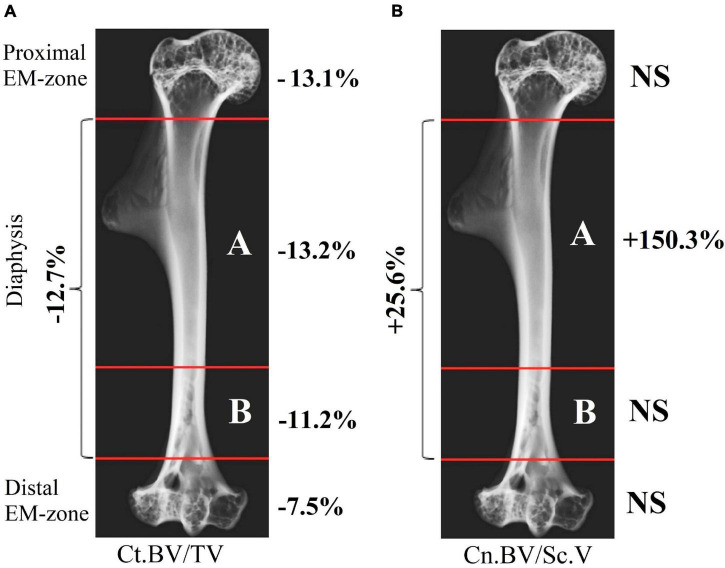
Quantitative estimation of cortical and cancellous bone volume fraction within different regions of the humerus after space flight. **(A)** Variation in the cortical bone volume fraction Ct.BV/TV; **(B)** Variation in the cancellous bone volume fraction (Cn.BV/Sc.V) (NS: no statistically significant differences were found between the control and flight groups).

**FIGURE 10:**
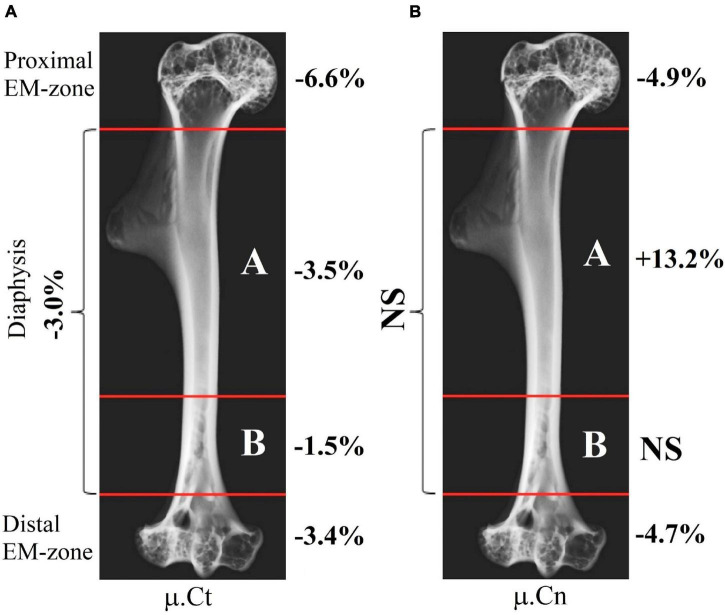
Quantitative estimation of X-ray linear attenuation coefficient within a different part of the humerus after space flight. **(A)** Variation of the linear attenuation coefficient in the cortical bone μ.*Ct*. **(B)** Variation of the linear attenuation coefficient in the cancellous bone μ.*Cn* (NS: no statistically significant differences were found between the control and flight groups).

Therefore, the cancellous bone volume in A and B bone regions in Figure 8B required a more accurate assessment. To achieve this, as a first step, the bone was segmented into the basic regions accounting for the variation of the attenuation coefficient in the cortical bone concerning the bone longitudinal coordinates (the procedure was described in the “Materials and Methods” section and is shown in Figure 3B). Then, an additional segmentation procedure was performed. It was based on the quantitative estimation of the cancellous bone volume fraction changing along the bone. This procedure resulted in the subdivision of the diaphysis into the proximal-A and distal-B (Figure 8B) regions. Note, that the bounder lines between the proximal EM-zone and the diaphysis and distal EM-zone and the diaphysis (Figure 3), have the same position after both the first-step segmentation and the additional segmentation procedure. Finally, the morphometric parameters of the proximal and distal regions of the diaphysis were estimated independently. The results are shown in Figures 9, 10 and Table 1. Images of trabecular compartments in proximal diaphysis (proximal zone—A) of the humerus are shown in Supplementary Section 2.

**TABLE 1:** Morphometric parameters of the humerus in control and flight groups after the experiment.

Index	Control group		Flight group		Difference, (%)	*p*-value
	(Mean	SD)	(Mean	SD)		
**Whole sample**						

Sa.Le, mm	15.9	0.68	15.6	0.33	−1.8	NS (0.178)
TV, mm^3^	39.3	3.06	38.1	2.16	−3.0	NS (0.264)
BV, mm^3^	22.1	1.73	19.5	1.12	−11.8	<0.001
BV/TV, %	56.3	2.10	51.2	2.09	−9.1	<0.001
μ.B, mm^–1^	1.131	0.023	1.080	0.026	−4.6	<0.001

**Proximal EM-zone**						

μ.*Ct*, mm^–1^	0.943	0.024	0.881	0.023	−6.6	<0.001
μ.*Cn*, mm^–1^	0.750	0.016	0.714	0.019	−4.9	<0.001
Ct.BV/TV, %	23.79	2.01	20.67	1.69	−13.1	<0.001
Cn.BV/Sc.V, %	18.78	1.11	18.74	1.16	−0.2	NS (0.937)

**Diaphysis**						

μ.*Ct*, mm^–1^	1.304	0.016	1.264	0.016	−3.0	<0.001
μ.*Cn*, mm^–1^	0.889	0.049	0.909	0.031	+2.3	NS (0.738)
Ct.BV/TV, %	69.0	2.84	60.23	2.64	−12.7	<0.001
Cn.BV/Sc.V, %	0.82	0.29	1.02	0.28	+25.6	0.073

**Diaphysis (proximal zone – A)**						

μ.*Ct*, mm^–1^	1.312	0.018	1.266	0.017	−3.5	<0.001
μ.*Cn*, mm^–1^	0.692	0.077	0.783	0.083	+13.2	0.008
Ct.BV/TV, %	68.49	3.32	59.46	2.72	−13.2	<0.001
Cn.BV/Sc.V, %	0.088	0.114	0.220	0.188	+150.3	0.015

**Diaphysis (distal zone – B)**						

μ.*Ct*, mm^–1^	1.278	0.026	1.259	0.021	−1.5	0.042
μ.*Cn*, mm^–1^	0.911	0.039	0.929	0.033	+2.0	NS (0.203)
Ct.BV/TV, %	70.18	2.96	62.30	2.99	−11.2	<0.001
Cn.BV/Sc.V, %	3.16	0.97	3.51	0.44	+11.1	NS (0.242)

**Mid-diaphysis**						

Tt.Ar, mm^2^	1.23	0.101	1.30	0.112	+5.6	NS (0.115)
Ct.Ar, mm^2^	0.98	0.066	0.92	0.05	−6.2	0.013
Ct.Ar/Tt.Ar, %	79.96	4.50	71.10	3.92	−11.1	<0.001
Ct.Th, mm	0.344	0.028	0.286	0.017	−16.9	<0.001

**Distal EM-zone**						

μ.*Ct*, mm^–1^	1.063	0.023	1.028	0.027	−3.4	0.002
μ.*Cn*, mm^–1^	0.836	0.047	0.796	0.024	−4.7	0.012
Ct.BV/TV, %	60.61	2.45	56.05	3.58	−7.5	0.001
Cn.BV/Sc.V, %	17.41	1.77	17.34	1.32	−0.4	NS (0.911)

**Distal epiphysis**						

Tb.Th, mm	0.078	0.008	0.070	0.003	−10.2	0.004
Tb.Sp, mm	0.258	0.038	0.227	0.022	−12.0	0.018
Tb.N, mm^–1^	3.69	0.46	4.08	0.37	+10.6	0.024

*NS, not significant; SD, standard deviation.*

The morphometric parameters of the bone computed by the developed method revealed new information on the humerus of the Mongolian gerbil. The cortical bone showed the variation of the bone volume fraction achieving the maximum value in the proximal part (proximal EM-zone, proximal diaphysis) and the minimum value in the distal EM-zone (Figure 9A). The change in the bone tissue of the cortical diaphysis was uneven. The bone loss in the proximal part of the diaphysis was higher compared with the rest of the diaphysis (Figure 9A). Moreover, bone loss in the proximal diaphysis was higher compared to the distal diaphysis (Figure 9A).

The evaluation of the cortical bone attenuation coefficient correlated with the abovementioned results (see Figure 10A). On the other hand, we detected a previously unobserved statistically significant increase by 13.2% in the attenuation coefficient of the cancellous bone within the proximal diaphysis (Figure 10B). The cancellous bone volume fraction (Cn.BV/Sc.V) increased by 150.3% in the proximal diaphysis. However, it remains unchanged over the proximal and distal EM-zones and the distal diaphysis (Figure 9B). We observed an increase of 11.1% in the cancellous bone volume fraction (Cn.BV/Sc.V) in the distal diaphysis after the flight (Table 1), but this change was not statistically significant, due to the relatively small size of the analyzed groups and the high interindividual variability of cancellous bone architecture. Therefore, future studies of post-flight alterations in the cancellous bone using an enlarged number of the control and flight group samples are required.

## Discussion

In our work, we investigated the skeletal system of gerbils in the first ever successful orbital experiment with these animals (Ilyin et al., 2008; Il’in et al., 2009; Soldatov et al., 2009). An essential issue in the study of adverse effects of long-term G_0_ on humans is a development of a relevant animal model based on the study of different species adapted to a different habitat and having different physiology and structure of the musculoskeletal system. On the other hand, a new animal model should maintain a correspondence to conventional animal models (Durnova et al., 2008).

Gerbils are social animals. They usually live in groups that typically consist of a parental pair, the most recent offspring, some older pups, and sometimes dominant female’s sisters. Intergroup differences in the average weight of gerbils before the experiment are closely related to the biological characteristics of these animals. Without family, gerbil can succumb to depression. That is why the unit of selection was the whole family (Il’in et al., 2009).

We observed weight loss in both flight and controls groups during the experiment. The reason could be a water-restricted diet when the only source of water for all groups of animals was the moisture contained in the food briquettes. Moreover, all gerbils were kept in relatively small cages, which served as an additional stress factor leading to weight loss in the groups.

According to our findings, the quantity of food varied between the groups. Basal control received more food compared to delayed synchronous control since there was not any feeder failure during the basal experiment. That is why gerbils from the basal control group lost less weight during the experiment (5.9%) compared to gerbils from the delayed synchronous control group (12.9%). However, we note that the imitation of the feeder failure in the group of delayed synchronous control began 4 days after the failure of the feeder in the flight group (see the “Materials and Methods” section). Therefore, gerbils from the delayed synchronous control group received more food compared to the flight group. In addition, flight animals received less food compared to both controls because the part of food remnants was removed from the cage together with excrements by the waste disposal system onboard (weight loss in the flight group was 28.3%).

To eliminate the discrepancy in the feeding regime of flight and delayed synchronous control groups (see the “Materials and Methods” section), an additional ground model experiment was carried out after the flight. The experiment accurately reproduced the failure of the feeder in the flight group. Twelve gerbils were kept in a replica of the flight habitat cage for 12 days, according to the spaceflight mission conditions. The average bodyweight of the animals was about 43.0 g before the model experiment and about 29.3 g after the model experiment. A weight loss of 31.8% in this group was comparable with the weight loss in the flight group. As a result, we can assume that the significant difference in average weight of the flight and delayed synchronous control animals are associated with a malfunction of the feeder (Il’in et al., 2009).

Furthermore, since no significant differences in humerus morphometry were found between the delayed synchronous control and basal control groups, we expect that the differences observed between flight and control groups would mainly be due to space flight factors, even if the initial differences in bodies mass, genetic differences between families, as well as small differences in ambient temperatures, have an impact on the result (Robbins et al., 2018).

In our research, we aimed to identify bone sites subjected to bone loss. The assumption about the site-specific effects of G_0_ on the humerus was based both on different morphology of the diaphysis, metaphysis, and epiphysis and on the consideration of specific mechanical loads in these bone sites.

Objective assessment of morphological changes in the musculoskeletal system of animals and humans is associated with a number of problems due to the complex morphological structure of the skeleton. Each region of the bone performs different functions to compensate for mechanical loads. Moreover, the neighboring regions with different architectures should smoothly merge while preserving the mechanical stability of the entire bone. As a result, functionally different regions having different spatial organizations often could not be associated with some clear histological boundary. This causes problems in a bone segmentation procedure and complicates the quantification of bone parameters. The density of the extracellular matrix in the bone reflects the functional specialization of the bone sites. Therefore, the regions with different bone densities can be delineated with a morphological boundary. The main problem in the region delineation procedure relates to the metaphysis, where bone density increases gradually.

Despite the distinct structural peculiarities of each zone of the bone, to our best knowledge, there was not before an explicit criterion for the bone division into the zones. We note that the development of an appropriate algorithm for the confident recognition of structurally distinct bone regions is one of the important tasks to be dealt with in biomedical space study.

The published results on post-flight micro-CT researches of bones in animal models are diverse. This may be partially related to the choice of the species and bones, with sex, age, and genetic features of the animals, as well as with the conditions of the space-flight (e.g., different housing and food) and micro-CT experiments (Cavolina et al., 1997; Turner, 2000; Tavella et al., 2012; Nagaraja and Risin, 2013; Keune et al., 2015; Berg-Johansen et al., 2016; Gerbaix et al., 2017; Giuliani et al., 2018).

On the other hand, an inaccurate experimental data processing procedure can have a negative impact on the correct results in data interpretation. In particular, this applies to different criteria and different anatomical landmarks for choosing an ROI in the bone (Yamashita et al., 2000; Almeida et al., 2006; Canciani et al., 2015; Keune et al., 2015; Berg-Johansen et al., 2016). As a rule, to assess the morphometric parameters of bones in micro-CT images, the ROI is selected in a semiautomated or manual way in each tomographic slice of the volume within the studied parts of the specimen. In the analysis of the cancellous region of the long bone (femur, tibia), the ROI in the metaphysis is usually selected, taking the epiphyseal plate as an anatomical reference. The ROI is selected with a small offset from the growth plate toward the diaphysis. To calculate the parameters of cortical bone, the ROI is usually set at mid-diaphysis. However, it was reported that the growth plate could be used as a reference as well (Salmon, 2019). In the studies on murine spinal segments (Berg-Johansen et al., 2016) ROI for trabecular bone was limited by the growth plate on the cranial end of the vertebra and extended 1 mm toward the diaphysis. To evaluate the morphometric parameters of the humerus in Keune et al. (2015) the cortical bone was segmented in the diaphysis, and the entire cancellous compartment was segmented in the distal epiphysis.

Analysis of local ROI in different bone regions without consideration of the general gross anatomy of the bone could lead to a potential inconsistency of study findings. In particular, without a clear definition of the boundaries between the bone regions, it would be difficult to reproduce the results.

We developed a numerical algorithm for a micro-CT study of the long bone, which gives the key criteria to delimitate the morpho-functional boundaries between the main bone regions. The developed algorithm uses cortical bone density as an evaluation criterion for the delineation of the diaphysis and proximal/distal EM-zones. The 3D analysis of the micro-CT images of the humerus bone presented here provides the main morphometric parameters of both cortical and cancellous tissues in all principal regions of the bone.

We found that bone volume fraction (BV/TV) in the gerbils of the flight group was 9.1% less than that in the control group. It could be a result of site-specific after-flight bone demineralization and occurred mostly in weight-bearing parts of the bone. Assuming this, postflight morphometric estimation has shown a significant mineral loss in the cortical part of the proximal EM-zone and proximal diaphysis. No statistically significant changes of volume fraction in the cancellous tissue of proximal and distal EM-zone were observed. A statistically significant increase in both cancellous bone volume and bone X-ray attenuation in the flight group was detected in the proximal part of the diaphysis. This may indicate that in response to a prolonged period of G_0_, bone minerals were not only excreted from the bone matrix through the circulatory system but also were partially redistributed from cortical to cancellous bone and deposited in the cancellous bone as a store of calcium. We also revealed a slight (4.7–4.9%) decrease in the linear attenuation coefficient (μ.*Cn*) in the cancellous bone of the distal and proximal EM-zones without statistically significant changes in volume fraction (Cn.BV/Sc.V). In the distal epiphysis, a decrease in the thickness of trabeculae (Tb.Th) of the cancellous bone by 10.2% was found, while the distance (Tb.Sp) between them decreased by 12.0%, and their number (Tb.N) increased by 10.6%. We assume that an uneven loss of the extracellular matrix occurred in G_0_. In this case, bone trabeculae in the distal epiphysis could split, form ruptures or holes in places of degradation. This could lead to an increase in the number of trabeculae and a decrease in the distance between them.

The cancellous bone composed of thin, less dense trabeculae tissue covers a broader surface area compared with the dense and compact cortical part of the epiphyses and diaphysis. Hence, bone resorption in G_0_ should be higher in the cancellous bone (Vico et al., 2000; Lang et al., 2004, 2017; Bloomfield et al., 2011; Nagaraja and Risin, 2013; Gerbaix et al., 2017; Maupin et al., 2019; Tominari et al., 2019). However, these results were reported mainly for the loaded bones of the hind limbs (Vico et al., 2000; Lang et al., 2004; Gerbaix et al., 2017). The humeral bones are less studied. Some authors reported no changes in the humerus after a 14-day space flight, (Keune et al., 2015) others reported significant changes in the bone after a 34-day space flight (Tominari et al., 2019). Since G_0_ can influence differently upon the different parts of the bone (Keune et al., 2015), the accurate and correct quantitative analysis of the cortical and trabecular regions of the proximal epiphysis, diaphysis, and distal epiphysis is a substantial issue in studies of spaceflight-induced bone loss.

Durnova et al. (2008) applied histomorphological methods to analyze the proximal tibial epiphyses of the Mongolian gerbils after a 12-day space flight. The humerus of the same gerbils we used here as an object of the investigation. According to Durnova et al. (2008), in the proximal tibial epiphyses, flight gerbils demonstrated the inhibition of bone growth in length and the development of osteopenia. The authors reported a decrease in the mass of cancellous bone and they associated it with inhibition of bone formation in G_0_. On the other hand, they underlined that the involvement of bone resorption in the development of osteopenia remains an open question. The number of trabeculae of secondary spongiosa was insufficient to prove the fact that a contemporary increase in bone resorption and inhibition of bone formation were due to an increase in the number of functional activity of osteoclasts (Durnova et al., 2008).

We did not observe similar changes in the humerus of flight Mongolian gerbils. On the one hand, we assume that, in both the humerus and tibia of space-flown gerbils, the prevailing process in bone calcium turnover could be the redistribution of the extracellular matrix within the bone rather than systemic bone loss. On the other hand, the observed results may be due to the differences in morphology, metabolism, and mechanical loading between the humerus and tibia.

Cavolina et al. (1997) and Keune et al. (2015) studied the skeletal system of the ovariectomized growing rats after a 2-week space flight. According to Cavolina et al. (1997), reported histomorphometric research on the rat’s tibia, a reduced periosteal bone formation with a reduction in the cancellous bone area and increase in bone resorption were observed in space-flown rats. Keune et al. (2015) have published micro-CT results on loaded and unloaded bones of the skeleton (including humerus) of the same rats. The study of the humerus has shown a G_0_-induced increase of marrow area in the diaphysis without significant effect on humerus length, bone area, bone mineral content, and bone mineral density. The authors noted that the impact of G_0_ on bone microstructure in ovariectomized rats was bone- and site-specific but it was not strictly correlated to load-bearing (Keune et al., 2015).

A reasonable explanation of the observed results can be based on the behavior of rodents in weightlessness. The analysis of the video-recording of gerbils in G_0_ suggests that floating gerbils often and actively use their forelimbs for movement (running, jumping), as was previously shown for mice (Cancedda et al., 2012; Ronca et al., 2019) and is known as a predominate use of hands for humans (Bloomfield et al., 2016). Human studies have shown that bone loss in the tibia after spaceflight was significantly greater than in radius (Bloomfield et al., 2016). It is likely that the resulting mechanical load leads to inhibition of osteopenia in the forelimbs. However, to prove this hypothesis, a quantitative analysis of video materials and a study of the humerus bones of gerbils using high-resolution micro-CT are required.

The resolution in micro-CT used here (pixel size is 9 microns) allows for morphometric analysis, especially of relatively thick cortical bone in the diaphysis. However, insufficient spatial resolution may provide incomplete information about the fine trabecular apparatus of the gerbils’ humerus. The trabecular thickness (Tb.Th) in the distal epiphysis of the humerus reported here was in the range 70–78 microns. We plan to carry out experiments using synchrotron radiation facilities and a voxel size of 1 um^3^ or less. This will provide additional information about the cancellous bone architecture. The use of high-resolution micro-CT would be of particular interest for the investigation of the cancellous compartment of the proximal diaphysis, where we supposed a notable after-flight effect on the cancellous bone.

## Conclusion

The study of the humerus of the Mongolian gerbil after the 12 days of space flight was carried out *via* micro-CT. A novel algorithm for an accurate delimitation of humerus bone regions has been developed. The differences between the flight and control groups in the cortical and trabecular zones of the specimen epiphysis and diaphysis were quantified. Evaluation of the bone parameters has shown a statistically significant decrease in both bone volume and bone X-ray linear attenuation coefficient in most regions of the humerus. The humerus mineral loss began from dense cortical areas; the most significant alteration was observed in the proximal EM-zone. Although no statistically significant changes in the cancellous bone volume fraction in proximal and distal EM zones were detected, a statistically significant increase in both cancellous bone volume fraction and bone attenuation coefficient was observed in the proximal part of the diaphysis. The data analysis suggests that in weightlessness, a remarkable part of the bone mineral content is actively involved in the bone response. Nonetheless, differences in initial body weight and genetic differences between families, as well as small fluctuation in ambient temperature, may have contributed to the results of the experiment. In this framework we presume, that in G_0_ calcium in bone is partially released into the circulatory system, and this determines the overall demineralization of the bone. Moreover, part of the mineral may redistribute from cortical to cancellous bone. We suggest that in-flight disorders in the calcium metabolism leading to enhanced calcium mobilization in the cancellous tissue are a bone response to weightlessness conditions.

## Data Availability Statement

The raw data supporting the conclusions of this article will be made available by the authors, without undue reservation.

## Ethics Statement

The animal study was reviewed and approved by Biomedicine Ethics Committee of the Russian Federation State Research Center – Institute of Biomedical Problems, Russian Academy of Sciences/Physiology Section of the Russian Bioethics Committee of Russian Federation National Commission for UNESCO.

## Author Contributions

YK: formal analysis, investigation, software, writing—original draft, review and editing. VG: investigation, resources, writing—original draft, review and editing. AB: software, methodology, formal analysis, writing—review and editing. DZ: investigation. AC: writing—review and editing. IB: validation, visualization, writing—original draft, and writing—review and editing. VA: conceptualization, project administration, and resources. SS: conceptualization, project administration, resources, and writing—original draft. All authors contributed to the article and approved the submitted version.

## Conflict of Interest

The authors declare that the research was conducted in the absence of any commercial or financial relationships that could be construed as a potential conflict of interest.

## Publisher’s Note

All claims expressed in this article are solely those of the authors and do not necessarily represent those of their affiliated organizations, or those of the publisher, the editors and the reviewers. Any product that may be evaluated in this article, or claim that may be made by its manufacturer, is not guaranteed or endorsed by the publisher.
