# Supplementary material for: Micro-CT Study of Mongolian Gerbil Humeral Bone After Prolonged Spaceflight Based on a New Algorithm for Delimitation of Long-Bone Regions
Source: Front Physiol. 2021 Dec 7;12:752893. doi: 10.3389/fphys.2021.752893 (PMC8688953; doi:10.3389/fphys.2021.752893)
Supplement: Supplementary file 1 [file Data_Sheet_1.docx]

***Supplementary Material***

**Supplementary Section 1**

**The trabecular bone morphometric parameters in the proximal epiphysis and metaphysis**

The calculation of the trabecular parameters in the proximal EM-zone (proximal/superior epiphysis & metaphysis) was performed. For this purpose, the proximal epiphysis and metaphysis were segmented, using the epiphyseal plate as an anatomical reference. The segmentation procedure was performed semi-automatically. The longitudinal section of the proximal part of humerus with segmented epiphyseal and metaphyseal subcortical volumes is shown in **Supplementary** **Figure 1**. Video demonstration of the segmentation results of the humerus is presented in the attached file (**Supplementary Video 1.avi**).


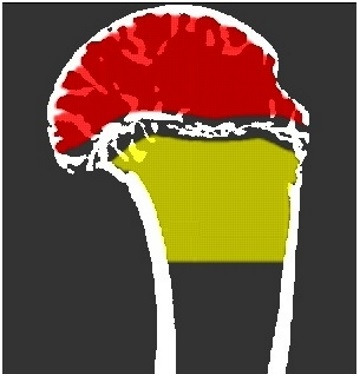


**Supplementary Figure 1.** Representative image of segmented proximal epiphyseal (red) and metaphyseal (yellow) subcortical volume, taking the epiphyseal plate as an anatomical reference.

The linear attenuation coefficient of cancellous bone ($\mu.Cn$), cancellous bone volume (Cn.BV), cancellous bone volume normalized by subcortical volume (Cn.BV/Sc.V – cancellous bone volume fraction) were calculated for proximal epiphysis and metaphysis. 3D model-independent algorithm [1] was used to calculate the bone morphometric parameters such as average trabecular thickness (Tb.Th), average trabecular separation (Tb.Sp), average trabecular number (Tb.N) in the proximal epiphysis and metaphysis.

To calculate the trabecular bone parameters Tb.Th, Tb.Sp, Tb.N we exclude the central part of the proximal metaphysis from consideration. In the proximal metaphysis of gerbils humerus the cancellous bone is mostly located near to the cortical part, while the cancellous bone amount in the central part of the metaphysis is extremely small or trabeculae may absent. The parameters Tb.Sp, Tb.N might be incorrect if the 3D model-independent algorithm for morphometric parameter estimation takes into account the central part of the metaphysis. In order to exclude the central part from consideration, the morphological operation "erosion" was used. Each tomographic slice of the metaphyseal subcortical volume was "eroded" until 3% of trabeculae volume compared to the total trabecular bone volume remained in the central part. Therefore, the ROI marked in red in **Supplementary Figure 2** contains 97% of trabeculae.

Three-dimensional micro-CT image of segmented cancellous bone in proximal metaphysis is presented in **Supplementary** **Figure 3.** The results of the calculations are presented in the **Supplementary Table 1**.


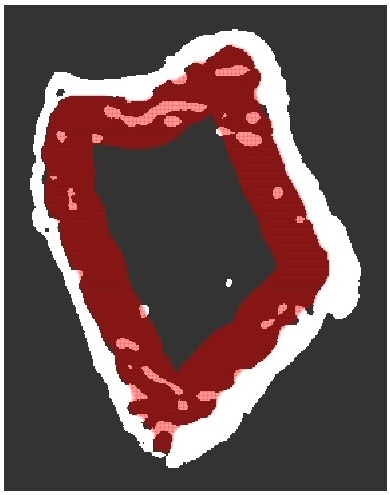


**Supplementary Figure 2.** Micro-CT binary cross-section of the proximal metaphysis of humerus. Red color marks an additionally segmented ROI in the subcortical volume of the proximal metaphysis for calculating the trabecular parameters Tb.Th, Tb.Sp, Tb.N.


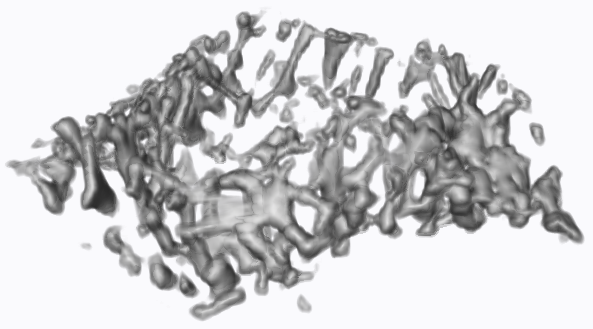


**Supplementary Figure 3.** Three-dimensional micro-CT image of segmented cancellous bone in proximal metaphysis of humerus.

**Supplementary Table 1. Trabecular bone morphometric parameters in control and flight groups**

| **Index** | **Control group** | | **Flight group** | | **Difference,%** | **p-value** |
| --- | --- | --- | --- | --- | --- | --- |
|  | **Mean** | **SD** | **Mean** | **SD** |  |  |
| **Proximal epiphysis** |  |  |  |  |  |  |
| Cn.BV, mm^3^ | 0.97 | 0.13 | 1.02 | 0.12 | +4.3 | NS (0.817) |
| Cn.BV/Sc.V, % | 17.50 | 1.66 | 17.78 | 1.50 | +1.6 | NS (0.647) |
| $\mu.Cn,$ mm^-1^ | 0.767 | 0.028 | 0.744 | 0.019 | -3.0 | 0.021 |
| Tb.Th, mm | 0.073 | 0.005 | 0.067 | 0.002 | -7.8 | <0.001 |
| Tb.Sp, mm | 0.279 | 0.024 | 0.254 | 0.024 | -8.8 | 0.016 |
| Tb.N, mm^-1^ | 3.36 | 0.22 | 3.73 | 0.37 | +10.8 | 0.008 |
|  |  |  |  |  |  |  |
| **Proximal metaphysis** |  |  |  |  |  |  |
| Cn.BV, mm^3^ | 0.238 | 0.057 | 0.242 | 0.038 | +1.6 | NS (0.487) |
| Cn.BV/Sc.V, % | 5.76 | 1.19 | 6.39 | 0.90 | +11.0 | NS (0.136) |
| $\mu.Cn,$ mm^-1^ | 0.733 | 0.022 | 0.687 | 0.011 | -6.2 | <0.001 |
| Tb.Th, mm | 0.063 | 0.004 | 0.054 | 0.002 | -14.1 | <0.001 |
| Tb.Sp, mm | 0.199 | 0.017 | 0.180 | 0.009 | -9.5 | 0.002 |
| Tb.N, mm^-1^ | 4.91 | 0.26 | 5.16 | 0.27 | +5.0 | 0.027 |

NS – Not Significant; SD – standard deviation.

**Supplementary Section 2**

**Trabecular compartment in proximal diaphysis (proximal zone - A) of humerus.**

A statistically significant increase in the cancellous bone volume fraction (Cn.BV/Sc.V) was detected in the proximal diaphysis (proximal zone - A) of the humerus.

A small number of trabeculae were observed in the proximal diaphysis (zone-A) near to the proximal EM-zone (**Supplementary Figure 4 A,C**), as well as in the deltoid crest (**Supplementary Figure 4 B,D**). The volume of trabecular bone in the both zones is approximately the same in flight and control groups of animals.

However, trabecular struts in the middle part of proximal diaphysis, detected mainly in the flight group (**Supplementary Figure 5**), led to a differences of the cancellous bone volume fraction (Cn.BV/Sc.V) in different groups of animals. Sample numbers for which trabecular struts were found in the middle part of proximal diaphysis: control group N 8, 9, 11, 15; flight group N 19 – 27. The reasons of trabecular struts growth in the middle part of the diaphysis are currently not clear and require additional research.


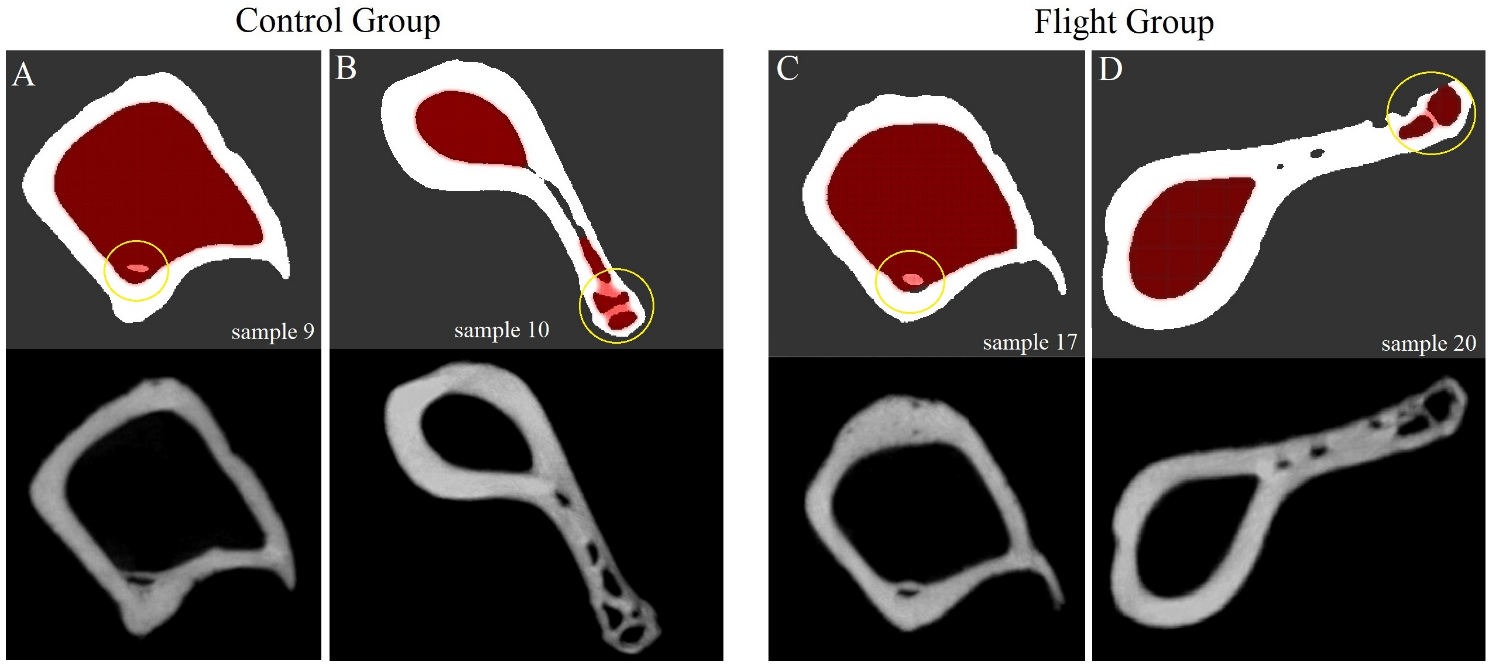


**Supplementary Figure 4.** Micro-CT cross-sections (top images) in the proximal diaphysis (zone A) of humerus. Areas containing trabeculae are indicated by yellow circles. Red color marks a subcortical volume (Sc.V) segmented for calculating the cancellous bone volume fraction (Cn.BV/Sc.V). Three-dimensional micro-CT images (bottom images) constructed of 20-50 layers near a cross-section.


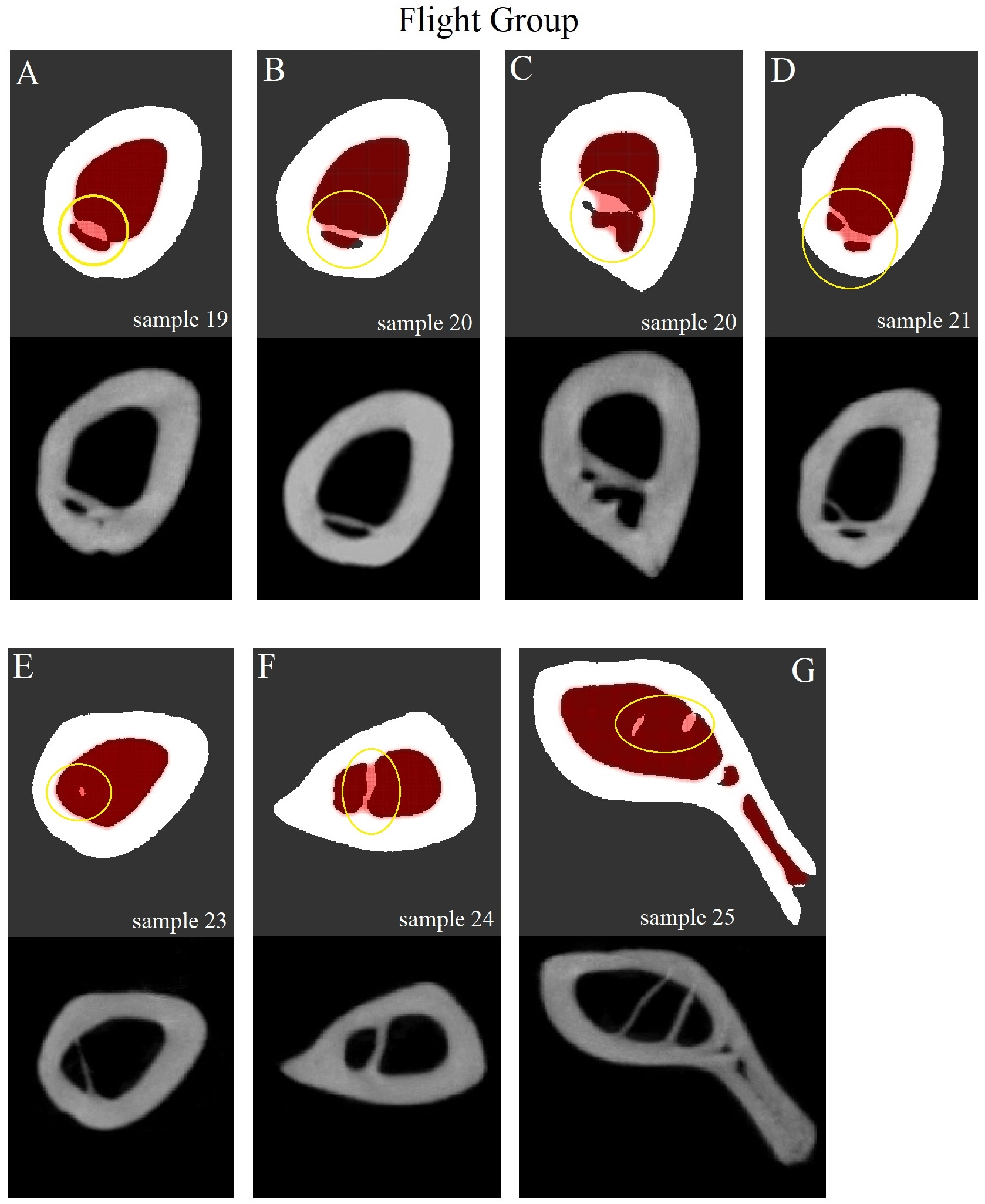


**Supplementary Figure 5.** Micro-CT cross-sections (top images) in the proximal diaphysis (zone A) of humerus. Areas containing trabeculae are indicated by yellow circles. Red color marks a subcortical volume (Sc.V) segmented for calculating the cancellous bone volume fraction (Cn.BV/Sc.V). Three-dimensional micro-CT images (bottom images) constructed of 20-50 layers near a cross-section.

**Supplementary Section 3**

**Results of calculation of Spearman’s correlation coefficients**

**Supplementary Table 2.** Spearman’s correlation coefficients between the post-flight body weight of animals and the major morphometric parameters of humerus bone for the control and flight groups. TV - volume of the whole examined sample, BV - bone volume, BV/TV - normalized volume index, μ.B - linear attenuation coefficient of bone.

|  | **Control group**  **gerbil's body weight**  (g) | | **Flight group**  **gerbil's body weight**  (g) | |
| --- | --- | --- | --- | --- |
| **Whole sample** | **R** | **p-value** | **R** | **p-value** |
| TV, mm^3^ | 0.616 | 0.0190 | 0.455 | 0.1377 |
| BV, mm^3^ | 0.416 | 0.1392 | 0.322 | 0.3079 |
| BV/TV, % | -0.141 | 0.6311 | -0.238 | 0.4568 |
| μ.B, mm^-1^ | -0.251 | 0.3871 | 0.063 | 0.8459 |

**References**

1. Hildebrand, T., & Ruegsegger, P. (1997). A new method for the model-independent assessment of thickness in three-dimensional images. Journal of Microscopy, 185(1), 67–75. doi:10.1046/j.1365-2818.1997.1340694.x
